# Health Tract, No. 248

**Published:** 1866-06

**Authors:** 


### WEIGHTS AND MEASURES

Convenient for sick and well and domestic purposes. If a dose of medicine for a man is sixty grains, then a one year old requires five ; 2 years, eight ; 3 years, ten ; 4 years, fifteen ; 7 years, twenty ; 14 years, thirty ; 20 years, 40.

Sixty drops make one teaspoonful, or one dram ; four teaspoonfuls make one tablespoon ; two tablespoons, an ounce ; two ounces a wine-glass ; four ounces a teacup or gill, or quarter of a pint ; sixteen ounces, one pint.

A French metre or measure of length, is in round numbers, thirty-nine inches ; the Litre, the measure of capacity in cubic inches, sixty-one. The gramme, the measure of weight, is sixteen and a half Troy grains. The killogramme is two pounds.

A box four inches long, four inches broad, and two and a quarter inches deep, holds one quart ; if four by four, and four and one eighth inches deep, it holds half a gallon ; if 8 by 8½ and eight inches deep, it holds one bushel ; if 24 by 16 and twenty-two inches deep, it holds one barrel. A convenient half bushel box is one foot square, and seven and a half inches high. As 2150½ cubic inches make a cubic foot ; any three dimensions of a box multiplied together and making 2150½ inches, measures a cubic foot. A box, a foot square and nearly fifteen inches deep (14 934-1000) holds one bushel. The solid contents of a bin, multiplied by four and divided by five, gives the number of bushels contained. A bushel lacks ten cubic inches, or one third of a gill, of being one and a half cubic feet.

A *Decoction* in medicine, is an ingredient boiled in water.

An *Infusion* is a medicinal leaf, bark, root, or wood, soaked in water hot or cold.

A *Mixture* is several liquid ingredients made into one.

A *Solution* is a solid, dissolved in a liquid, as sugar in water.

A *Saturated Solution* is when the liquid will dissolve no more of that solid, and the undissolved part falls to the bottom.

A *Tincture* is the strength of any substance withdrawn from it, by being soaked in alcohol or any other spirit. Alcohol is the foundation of all spirits, it is the principle which causes drunkenness ; its constituents are found in all vegetable substances, and has different names according to the substance out of which it is made, thus, when made from grain, as wheat, rye, or corn, it is called whiskey ; if from grapes, it is Brandy ; if from sugar cane, it is rum. Gin is whiskey flavored with the juniper berry. Bourbon whiskey is made from corn, or rye, in copper stills, but it is said that the same materials, managed in the same way will not make the same article, except in or near Bourbon County.

All wines are radically alike, made of different materials, but causing intoxication according to the amount of alcohol in them, and without which principle they would all fall into disuse.

A cubic foot of water weighs 62½ lbs.; of seasoned wood 40 lbs.; of coke 50 lbs.; of coal 75 lbs.; of brick work 95 lbs.; of sandstone 140 lbs.; of granite 180 lbs.; of cast iron 450 lbs.; of wrought iron 480 lbs.

